# Technology-Supported Guidance Models Stimulating the Development of Critical Thinking in Clinical Practice: Mixed Methods Systematic Review

**DOI:** 10.2196/37380

**Published:** 2022-06-07

**Authors:** Jaroslav Zlamal, Edith Roth Gjevjon, Mariann Fossum, Marianne Trygg Solberg, Simen A Steindal, Camilla Strandell-Laine, Marie Hamilton Larsen, Andréa Aparecida Gonçalves Nes

**Affiliations:** 1 Department of Bachelor Education in Nursing Lovisenberg Diaconal University College Oslo Norway; 2 Department of Health and Nursing Sciences University of Agder Kristiansand Norway; 3 Department of Postgraduate Studies Lovisenberg Diaconal University College Oslo Norway; 4 Faculty of Health Studies VID Specialized University Oslo Norway; 5 Faculty of Health and Welfare Novia University of Applied Sciences Åbo Finland

**Keywords:** critical thinking, guidance models, technology, nursing education, clinical practice

## Abstract

**Background:**

Nursing education has increasingly focused on critical thinking among nursing students, as critical thinking is a desired outcome of nursing education. Particular attention is given to the potential of technological tools in guiding nursing students to stimulate the development of critical thinking; however, the general landscape, facilitators, and challenges of these guidance models remain unexplored, and no previous mixed methods systematic review on the subject has been identified.

**Objective:**

This study aims to synthesize existing evidence on technology-supported guidance models used in nursing education to stimulate the development of critical thinking in nursing students in clinical practice.

**Methods:**

This mixed methods systematic review adopted a convergent, integrated design to facilitate thematic synthesis. This study followed the guidelines of the *Joanna Briggs Institute Manual for Evidence Synthesis*.

**Results:**

We identified 3 analytical themes: *learning processes implemented to stimulate critical thinking*, *organization of the learning process to stimulate critical thinking,* and *factors influencing the perception of the learning process*. We also identified 4 guidance models, all based on facilitator or preceptorship models using tailored instructional or learning strategies and one or several technological tools that were either generic or custom-made for specific outcomes. The main facilitators of these technology-supported guidance models were nurse educators or nurse preceptors, and the main challenges in using technology-supported guidance models were the stress associated with technical difficulties or increased cognitive load.

**Conclusions:**

Although we were able to identify 4 technology-supported guidance models, our results indicate a research gap regarding the use of these models in nursing education, with the specific aim of stimulating the development of critical thinking. Both nurse preceptors and nurse educators play a crucial role in the development of critical thinking among nursing students, and technology is essential for such development. However, technology-supported guidance models should be supervised to mitigate the associated stress.

**International Registered Report Identifier (IRRID):**

RR2-10.2196/25126

## Introduction

### Background

The increasing complexity of modern health care demands not only a new kind of thinking among nurses but also a complex set of skills and competencies [1]. Although earlier nursing education focused on building knowledge [2], modern nursing education to teach highly professional nurses needs to shift its focus to nurses’ ability to combine various skills and competencies that demand critical thinking [3].

Critical thinking can be understood as a manifestation of a set of dynamic skills that are purposeful and self-regulatory [4]. A critically thinking nurse is creative, flexible, and open-minded [5] and is able to question established assumptions [6]. To acquire a deeper understanding of a situation, a critically thinking nurse considers its context [7] and exhibits the ability to think logically, seek information, and transform knowledge into actions [8]. Hence, critically thinking nurses are highly skilled professionals [7]. Despite the importance of critical thinking in nursing practice, concerns have been raised as to whether newly graduated nurses possess the necessary level of critical thinking skills [9-11]; therefore, the means by which nursing students become critical thinkers has become a recurring topic in nursing education [12].

Traditionally, clinical practice during nursing education has been an important learning context in becoming a highly skilled professional nurse and, as such, also a critical thinker. In European countries, learning has mostly been facilitated through a guidance model in which registered nurses as nurse preceptors provide continuing guidance and nurse educators maintain oversight of the learning process [13]. A guidance model is a distinct way of organizing clinical practice for nursing students, involving predefined tasks, procedures, and guidelines. According to these models, nursing students are guided in clinical practice to achieve learning outcomes [14].

The introduction of technological tools has transformed nursing education in many ways and facilitated flexible new approaches in the education of nursing students [15]. Previous research has questioned whether technology can be used to facilitate the development of critical thinking [16]. The use of tailored technological tools in nursing education seems still to be somewhat limited [17], but earlier research has shown that mobile-based learning may support the development of knowledge and nursing students’ skills both outside and within clinical practice [18]. Technology-supported guidance models draw on the principle of integrating technological tools into guidance models and the use of technological tools to increase knowledge and improve attitudes and learning outcomes [19].

Previous systematic reviews have focused on the effectiveness and obstacles of teaching strategies in the development of critical thinking, with or without the use of technology, but not in the context of clinical learning and guidance [20-24]. Other systematic reviews have focused on the use of mobile technology in nursing education but without an explicit focus on critical thinking or clinical learning and guidance [18,25,26].

In these reviews, some of the strategies identified to support the development of critical thinking were problem-based learning, concept mapping, simulation, narrative pedagogy, critical reading and writing, videotaped vignettes, web-based animated pedagogical agents, reflective writing, grand round strategies, videodisc systems, and evidence-based courses [20-24].

The identified obstacles and challenges in supporting the development of critical thinking include learning and educational culture, language barriers, lack of a common understanding of the term *critical thinking*, educators’ beliefs and knowledge, and attitudes about critical thinking [23].

No previous systematic review has combined the focus on critical thinking and its development with the use of technology as part of a guidance model in the context of clinical practice. For this purpose, we selected a mixed methods systematic review. A traditional systematic review would provide us with findings that could answer, for example, the effect of interventions; however, adding qualitative data to a systematic review and conducting a mixed methods systematic review enables us to explore a body of literature on both qualitative and quantitative approaches. This provides us with the possibility of examining research gaps and answering multiple research questions. As such, a mixed methods approach may increase the impact and use [27].

### Objectives

This review aimed to synthesize existing evidence on a range of guidance models supported by technology and enhance nursing students’ critical thinking during clinical practice.

### Research Questions

The research questions are as follows:

1. Which technology-supported guidance models are used to stimulate the development of critical thinking in the context of clinical practice in nursing education?
2. What are the challenges and facilitators of such technology-supported guidance models?

## Methods

### Design

This mixed methods systematic review adopted a convergent integrated design following the guidelines outlined in the *Joanna Briggs Institute* (JBI) *Manual for Evidence Synthesis* [28]. The review is reported according to the PRISMA (Preferred Reporting Items for Systematic Reviews and Meta-Analyses) 2020 checklist [29], as described in Multimedia Appendix 1. Deviations from the published protocol [30] are summarized in Multimedia Appendix 2 [31-35].

### Ethics Approval

This study is exempt from institutional review board evaluation [28].

### Eligibility Criteria

The eligibility criteria are elaborated in Textbox 1.

**Inclusion and exclusion criteria [30].**

**Inclusion criteria**

- Study population: preregistration nursing students or undergraduate nursing students
- Phenomenon of interest: technological tools used in clinical practice, technology-assisted guidance models, technology-supported guidance models, guidance models, mentoring, tutoring, preceptorship in clinical practice, or clinical educational models
- Context: clinical practice in hospitals, nursing homes, community health care, or other health care institutions and settings
- Type of study: qualitative, quantitative, and mixed methods studies using experimental, quasi-experimental, or nonexperimental design published in peer-reviewed journals
- Type of outcome: critical thinking, clinical decision-making, analytical thinking, creative thinking, problem solving, reflective thinking, diagnostic reasoning, and clinical judgment

**Exclusion criteria**

- Study population: nursing students studying at the master’s or graduate level; postregistration nursing students; student paramedics; students of midwifery, physiotherapy, or occupational therapy; medical students; and dental students
- Phenomenon of interest: technology-assisted guidance models; clinical educational models; guidance models; mentoring, tutoring, or preceptorship outside clinical practice in clinical laboratories or as a preparation for clinical practice; and simulation or technology use in conjunction with simulation
- Context: outside clinical practice, such as in classes for preparation for clinical practice, simulation sessions, and training in a clinical laboratory
- Type of study: any type of systematic or nonsystematic review, non–peer-reviewed articles, conference proceedings, comments or opinion articles, official guidelines, national nursing curriculums, editorials, abstracts, and doctoral theses
- Type of outcome: all outcomes other than those mentioned in the inclusion criteria

### Main Outcome

As previously published in the study protocol of this mixed methods systematic review [30], the primary outcome is critical thinking according to the definition of Facione [4], as well as synonyms of the term *critical thinking* as outlined in Textbox 1.

### Search Strategy

The review team chose the initial terms suitable for building a search strategy. Using Medical Subject Headings, CINAHL headings, and subject terms, a research librarian (Fredrik Solvang Pettersen), the first author (JZ), and the last author (AAGN) constructed a search strategy for MEDLINE and CINAHL. The search strategy was tested in MEDLINE and CINAHL, peer reviewed by a second research librarian (Mia Ølnes), and then further used in CINAHL, Cochrane Trials, Embase, ERIC, MEDLINE, PsycINFO, and Web of Science. The search strategy has been previously published [30] and is presented in Multimedia Appendix 3. In addition, the first (JZ) and last author (AAGN) conducted forward and backward citation searches. It was not feasible to conduct searches of the gray literature because of the lack of an accepted standard method to conduct such searches [36].

Database searches were performed on October 21, 2020. Database searches were updated on December 3, 2021.

### Data Management

Rayyan (Rayyan Systems Inc) [37] used a web-based tool to facilitate the screening process. We used the Paperpile (Paperpile, LLC) [31] web-based tool for record storage and management.

### Selection Process

On the basis of the inclusion and exclusion criteria, titles and abstracts were screened independently by pairs of authors [38] (AAGN and JZ, ERG and MF, MHL and CSL, and SAS and MTS). The first author uploaded the full-text articles to the Notion (Notion Labs, Inc) [39] web-based tool, enabling other authors to access them. The pairs of authors (AAGN and JZ, ERG and MF, MHL and CS-L, and SAS and MTS) then independently assessed the full-text articles and included or excluded them based on the eligibility criteria. We encountered uncertainty regarding the selection of some of the full-text articles, which were discussed with the team in question and the first and last authors. The final decision was made through consensus.

### Assessment of Methodological Quality

We appraised the methodological quality of the included studies using the JBI Critical Appraisal Tool checklist for qualitative research [40] and quasi-experimental studies [41]. For mixed methods studies, we used the Mixed Methods Appraisal Tool [42]. This process was conducted independently by the pairs of authors (AAGN and JZ, ERG and MF, MHL and CS-L, and SAS and MTS). We included all studies in the data extraction and synthesis, regardless of the results of the assessment of methodological quality.

### Data Extraction and Data Items

Using the standardized JBI Mixed Methods Data Extraction Form and using a convergent integrated method [28], the pairs of authors (AAGN and JZ, ERG and MF, MHL and CS-L, and SAS and MTS) extracted data from the included studies. We included data on the country of origin, year of publication, population, phenomenon of interest, type of study, methods, context, period, outcomes, percentages, averages, significant and nonsignificant results, and themes and subthemes [28].

### Thematic Synthesis

We adopted a thematic synthesis approach, which Thomas and Harden [43] regarded as founded upon thematic analysis [43]. The first author (JZ) uploaded the extracted textual data to the MAXQDA tool (VERBI GmbH) for further analysis [32], read the textual data, and used color marking to identify the data or concepts present. Quantitative data were transformed to qualitative data through the process of qualitization of data, qualitizing the data through textual descriptions of the quantitative data [28]. Following this process, the first author (JZ) conducted line-by-line textual coding, in which, text was segmented and initial codes were developed [43,44]. At this stage, textual data were not related to the research questions. Subsequently, the first author (JZ) reread the textual data, assigned codes, and reviewed them in relation to the research questions. At this stage of the synthesis process, the codes were combined and reduced and a codebook with code definitions was created. The first (JZ) and last (AAGN) authors then individually coded the text segments and jointly reviewed the results, looking for discrepancies or necessary adjustments. The codes were then further reduced and combined. Through this process, we developed descriptive themes that were closely related to the original text segments [43]. In the next step of the synthesis process, we developed analytical themes to generate new insights [43]. In total, 110 text segments were coded with 80 initial codes and subcodes, with their descriptions. The final number of codes was 14.

The completed codebook and text segments were sent to all the coauthors (ERG, MF, MHL, CS-L, SAS, and MTS), who also individually coded the text segments with the provided codebook. On the basis of this coding, the intercoder reliability was calculated [38] using the DataTab (DATAtab e.U.) [45] statistical tool, which yielded a Fleiss κ of 0.25 on individual codes, indicating fair agreement (0.21-0.40) [46]. After this process, we identified the need for further interpretation, abstraction, and combination of codes and themes. The first (JZ) and last (AAGN) authors conducted a final review of themes and codes relative to the original text segments and further combined and reduced the descriptive and analytical themes. A new intercoder reliability was calculated, which yielded a Cohen κ of 0.49 on individual codes, indicating moderate agreement (0.41-0.60) [47]. With regard to the descriptive themes, Cohen κ was 0.54, also indicating moderate agreement (0.41-0.60) [47]. On the analytical themes, Cohen κ was 0.62, indicating substantial agreement (0.61-0.80) among the coders [47]. The final results comprised 7 descriptive themes and 3 analytical themes. Table 1 provides a detailed overview of the results of intercoder reliability calculations. An example of the coding process is presented in Table 2.

**Table 1:** Fleis κ and Cohen κ of individual codes, descriptive themes, and analytical themes.

	On individual codes (intercoder reliability among all authors)	On individual codes (intercoder reliability between the first and last authors)	On descriptive themes (intercoder reliability between the first and last authors)	On analytical themes (intercoder reliability between the first and last authors)	Asymptomatic SE	95% CI
Fleiss κ	0.25	N/A^a^	N/A	N/A	0.02	0.21-0.29
Cohen κ	N/A	0.49	N/A	N/A	0.06	0.37-0.61
Cohen κ	N/A	N/A	0.54	N/A	0.07	0.40-0.68
Cohen κ	N/A	N/A	N/A	0.62	0.07	0.48-0.76

^a^N/A: not applicable.

**Table 2:** Example of the coding process.

Analytical themes	Descriptive themes	Code (identifier)	Text segment
Learning processes implemented to stimulate critical thinking	Learning	Learning activities (LA)	The students explained the procedures and nursing skills they had learnt.
Organization of the learning process to stimulate critical thinking	Help and support	Mentoring (ME)	The lecturers avoided teaching the students on the forum and instead tried to give the students possibilities to solve the problems themselves. Giving support to think, compare, and reflect the students’ nursing actions.
Factors influencing the perception of the learning process	Technological tools	Advantages of technological tools (ATT)	[The] student and instructor said that they greatly benefited from the use of the mobile e-based system for clinical practicums.

## Results

### Search and Selection Process

We identified 9553 records, of which, after removing 5317 (55.66%) duplicates, we screened the titles and abstracts of 4236 (44.34%) records. A total of 1.68% (17/9553) of reports were assessed for eligibility, and 0.04% (4/9553) of studies were included.

We identified 8 records through forward and backward citation searches. We retrieved these records and accessed them for eligibility; however, none were eligible for inclusion. We did not contact any other researchers in this field.

**Figure 1:**
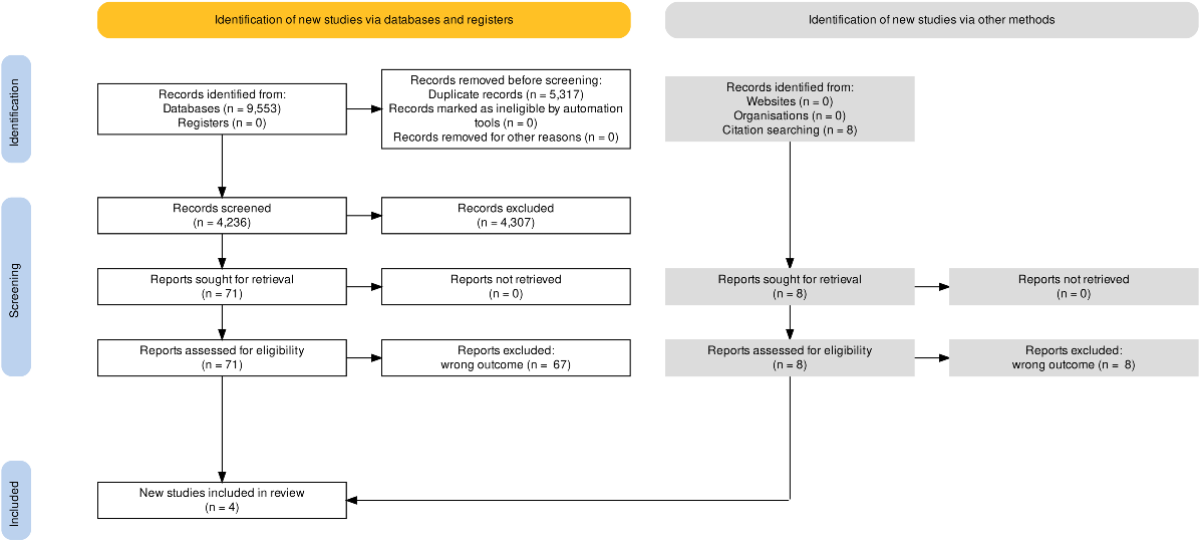
PRISMA (Preferred Reporting Items for Systematic Reviews and Meta-Analyses) flow diagram.

Figure 1 provides a detailed overview of the selection process and the reasons for exclusion.

### Study Characteristics and Methodological Quality

The studies included in this review (N=4) were conducted in Taiwan (n=3, 75%) [48-50] and Finland (n=1, 25%) [51]. Their sample sizes ranged from 8 to 64 [48-51], and their participants were undergraduate nursing students in clinical practice in community health practice (n=64), psychiatric nursing (n=18), and surgical theaters (n=25) [48-51].

All the studies (N=4) used the organized guidance of nursing students in a procedural setup, but the guidance models were poorly described in all the articles, which lacked detailed descriptions of both guidance procedures and student follow-up and cooperation between nurse preceptors and nurse educators [48-51].

The technological tools used in the studies included web-based discussion forums (1/4, 25%), mobile devices (1/4, 25%), e-portfolio systems (1/4, 25%), and e-book systems (1/4, 25%).

A description of the theoretical framework was provided in 2 studies [49,51].

Lai and Wu [48] measured critical thinking among participants using an author to develop a competency scale that included critical thinking and was a student- and instructor-assessed scale. Wu et al [50] also evaluated critical thinking on a scale that authors called “The seven dimensions of learning effectiveness.” No information was provided regarding the characteristics of the scales or the scoring methods.

All included studies (N=4) either lacked information, such as a description of the design and methods, or provided an unclear statistical analysis or unclear interpretation of the results [48-51]. Multimedia Appendix 4 [48-51] provides a detailed overview of the characteristics of the studies. The assessment of methodological quality is provided in Multimedia Appendix 5-8.

### Results Related to the Research Questions

#### Overview

To answer the research questions, the results were organized on the basis of the analytical themes presented in Table 3: *learning processes implemented to stimulate critical thinking*, *organization of the learning process to stimulate critical thinking,* and *factors influencing the perception of the learning process*.

The first research question aimed to identify technology-supported guidance models used to stimulate the development of critical thinking in the context of clinical practice in nursing education. All participants in the selected studies [48-51] received guidance during clinical practice through lecturers, nurse preceptors, or both, and the guidance was supported by technological tools. Table 4 provides a detailed overview of the organization of guidance and the technological tools used to support guidance.

**Table 3:** Comprehensive overview of analytical themes, descriptive themes, codes, and their definitions.

Analytical and descriptive themes		Code (identifier)	Code definition	Study
**Learning processes implemented to stimulate critical thinking**				
	Teaching	Instructional strategies (IS)	A teacher’s overall approach that facilitates the learning process and includes various teaching activities, strategies, styles, and training; includes time allocated for learning	[49,51]
	**Learning**			
		Learning activities (LA)	Activities targeted toward the learning process that involve technology and learning from one’s own and others’ experiences, discussions, or reflections	[49,51]
		Learning results (LR)	The effect of diverse approaches and guidance on the process of learning	[49-51]
		Learning strategy (LS)	Approaches that facilitate diverse learning styles without the use of technology	[49,51]
		Learning motivation (LM)	The motivation to learn; a personal drive to learn and acquire knowledge	[50,51]
	Professional knowledge	Knowledge construction (KC)	The process of evaluating, adding, explaining, transforming, and summarizing information, including self-awareness of what one does not know as well as reflection, self-judgment, self-observation, and consciousness of one’s own needs	[48,49,51]
	Professional skills	Competence (C)	The set of skills and abilities by which one connects theoretical knowledge with practice and understands context; includes both acquired and improved competence	[48,50,51]
**Organization of the learning process to stimulate critical thinking**				
	Technological tools	Use of technological tools (UTT)	Various uses of diverse technological tools to showcase one’s work, write daily journals, submit assignments, search for answers, complete daily tasks, access necessary information, and assess learning; technological tools serving as cognitive tools; use of discussion forums	[49,51]
	**Help and support**			
		Mentoring (ME)	The process of giving students the opportunity to solve problems and of providing support for thinking and reflecting on their actions	[51]
		Supervision (SUP)	The process of continuous supervision	[51]
		Peer support (PSU)	Sharing learned knowledge, creating dialogue and conversation, and understanding and encouraging one another	[51]
**Factors influencing the perception of the learning process**				
	**Technological tools**			
		Technical problems (TP)	Technical problems that make the use of technological tools challenging	[48]
		Advantages of technological tools (ATT)	Positive experiences of using technological tools and descriptions of their advantages	[48-50]
		Stress (ST)	Stress in relation to learning or using technological tools	[48,50]

**Table 4:** Organization of guidance and technological tools used in guidance.

Study	Organization of guidance	Technological tools used in guidance
Mettiäinen and Vähämaa [51]	Supervision organized in a web-based discussion forum in which guidance was provided by nurse educators with additional attention to peer support	An online discussion forum on the Moodle platform (a learning management system)
Lai and Wu [48]	A clinical e-portfolio system supervised by nurse educators	An e-portfolio system running on mobile netbooks
Lai and Yen [49]	Guidance provided by nurse educators, both with and without the use of technological tools	A mobile voice recording app, various evaluation apps, videos, mobile devices, and a web-based learning platform
Wu et al [50]	An e-book system in conjunction with guidance from nurse preceptors and nurse educators	A web-based e-book system

#### Learning Processes Implemented to Stimulate Critical Thinking and Organization of the Learning Process to Stimulate Critical Thinking

With regard to our primary outcome of critical thinking, the results showed that critical thinking was described in diverse terms in the selected studies. Mettiäinen and Vähämaa [51] refer directly not only to the term critical thinking but also to reflective thinking, whereas Wu et al [50] refer to both critical and creative thinking and problem solving. Lai and Wu [48] and Lai and Yen [49] also use the term critical thinking.

Technological tools were not used on their own but jointly with varied instructional and learning strategies and activities to stimulate the development of critical thinking. Among such strategies were discussion [51], demonstration [49], dividing students into groups to tailor learning, compulsory participation and preparation of students for activities, giving students space and time to learn, and supporting students through their learning while motivating them to share their experiences [51].

Other strategies include checking factual information against theory [51], viewing one another’s assignments [48], comparing diverse learning strategies, posting and reading comments in a web-based environment [51], and observing patients’ states [49]. During these activities, improvements in the ability to reflect [49,51] and improved theoretical knowledge and practical skills were observed [48]. Students also experienced improved self-satisfaction and became aware of their own feelings, learning needs, and areas where their skills needed improvement [48,50,51].

To support the development of critical thinking, lecturers supported students in various ways, such as by providing feedback [50] and giving time and opportunities for students to find their own solutions [51]. The lecturers avoided teaching the students and gave them opportunities to solve problems themselves. The lecturers also provided comments and feedback and supported the students’ thinking, reflection, and discussion. The lecturers followed the students’ discussions and provided active and continuous supervision [51]. The students also found support from their peers, with whom they could share knowledge, feelings, conversations, encouragement, and understanding [51].

Lai and Wu [48] measured critical thinking as a part of competency among participants, as assessed by instructors and showed increased scores from 2.7 at week 1 to 4.3 at week 3 (*P*<.001). Wu et al [50] did not provide any results for critical thinking in their evaluation of “The seven dimensions of learning effectiveness.”

#### Factors Influencing the Perception of the Learning Process

The second research question aimed to identify the challenges and facilitators of technology-supported guidance models. In the included studies, we found that some guidance models used custom-made technological tools (such as bespoke software) that were created or adapted for the context [48,50,51], whereas others used mobile devices in a more generic way [49]. In addition, some guidance models used only one technological tool, whereas others incorporated several technological tools simultaneously, as noted in Table 4. All the technological tools described in the chosen studies at least partially required access to the internet to work for their intended purpose [48-51]. Some technological tools required that users connect to the internet at home [51], whereas others used the available internet access in clinical practice [48-50].

The students deemed technological tools to be beneficial facilitators of learning in clinical practice [48]. These tools facilitate reflection and decision-making [50]; serve as additional learning resources [48]; facilitate discussions [50,51]; and improve competencies, patient care, and interactions with instructors [48]. Technological tools also gave students opportunities to showcase their own work [50], achieve clinical practice objectives [50], write daily reflective journals, and take notes of important information [49]. They also facilitated patient assessment, writing and submitting assignments, summarizing information, and recording instructors’ demonstrations [50]. The students who used technological tools in clinical practice scored better on evaluation instruments than their counterparts who did not use the tools [50].

However, technology-supported guidance models were also associated with stress, which was identified in relation to the challenges or mental load of students in a technology-supported guidance model. Some students experienced an increased mental load related to spending more energy and mental resources on tasks in clinical practice than those who did not receive guidance involving technology [50]. Other students experienced stress related to technical challenges that hindered the use of technological tools, such as setting up a wireless internet connection in clinical practice [48].

## Discussion

### Principal Findings

This review synthesizes the existing evidence on a range of guidance models supported by technology to enhance nursing students’ critical thinking during clinical practice. However, of the 71 reports assessed for eligibility, only 4 (6%) were eligible for inclusion, which may point to a research gap. The topic of technology-supported guidance models in conjunction with critical thinking and in the context of nursing students’ clinical practice appears to be an underresearched area.

In all the included studies, guidance and technological tools were set up and organized within a guidance model, a framework with an organized, predefined set of procedures [14]. We identified diverse types of technology-supported guidance models in which nurse preceptors, nurse educators, or both provided guidance with the support of one or more technological tools. Although none of the studies offered detailed descriptions of the guidance model, all the guidance models we identified were either preceptor and facilitator or preceptor based [52]. The facilitation- or preceptor-based guidance model, in which nursing students, nurse preceptors, and nurse educators cooperate in the guidance process, is common in European countries. The main advantage of this approach is the provision of mutual support and exchange of knowledge, which ensures that nursing students develop the necessary competencies and achieve learning outcomes [53]. However, the degree of cooperation may vary, and cooperation may be challenging [53,54].

We also found that the guidance of nursing students in these technology-supported guidance models occurs in 2 distinct ways. In one instance, guidance was provided by a nurse preceptor in clinical practice without access to or the use of technological tools, whereas guidance was simultaneously provided by nurse educators with the support of such tools. However, if the common guidance models are facilitation- or preceptor-based [53], nurse educators and nurse preceptors do not have access to or use the same technological tools, which may exacerbate division and result in less cooperation and support. In other instances, guidance with access to and support from the same technological tools was provided by both nurse preceptors and nurse educators.

We found that only half of technology-supported guidance models were based on a theoretical framework [49,51]. If we regard such models as interventions, it is necessary to base the intervention on a theoretical framework that can provide understanding and important insights on how a guidance model works and creates change [55].

This systematic review also found that technology-supported guidance models include diverse instructional and learning strategies, including reflection, discussion, and demonstration. Thus, our results confirm the findings of previous research, which showcase a plethora of approaches to support the development of critical thinking [20-24].

Regardless of the approach to critical thinking, it is regarded as something that can be learned [12]. Our findings identified learning activities targeted at critical thinking, such as comparing and contrasting, observational learning, discussion in an e-environment (ie, a discussion forum), and project-based learning. According to Krishna et al [56], students can gain a deeper understanding and develop critical thinking by comparing different concepts or processes; with regard to observational learning, it is assumed that observation contributes to learning through behavior change [57]. Wang et al [58] and Puig et al [59] pointed out that technological tools, such as e-environments, bring advantages that enhance the learning of critical thinking skills, as students in an e-learning environment can easily discuss, collaborate, or practice diverse skills. As Lee et al [21] noted, there is no universal agreement on the most suitable approach to developing critical thinking. However, this diversity of approaches may reflect that critical thinking is a multidimensional concept; as such, the development of critical thinking can be approached in various ways [12].

In our findings, the students perceived technological tools as essential to clinical practice; they might be used for many purposes, such as a knowledge database, sharing of experiences, or as knowledge or communication tools [48-51]. Contrary to our results, Lee et al [25] found no evidence that technological tools, such as mobile technology, support the development of knowledge or skills.

We identified nurse educators as the main facilitators of technology-supported guidance models. Nurse educators have facilitated these guidance models by supporting students in various ways, such as allowing time for students to find their own solutions [51] and providing situational feedback [48,50]. Nurse educators also avoided teaching the students and gave them opportunities to solve problems themselves. Nurse educators provided comments and feedback and supported students in thinking, reflection, and discussion.

Nurse educators also followed the discussions and provided active and continuous supervision [51]. These findings on the contributions of nurse educators have been confirmed in previous research [53,54] and point to the fact that technological tools in guidance models cannot be introduced on their own but require oversight, as well as support and mentoring by nurse educators, and peers may be a valuable addition in facilitating technology-supported guidance models. Students perceive their peers as facilitators who provide support by sharing knowledge and feelings, engaging in conversation and encouraging and understanding one another [49,51]. The positive influence of peer support and interaction has also been confirmed in previous research [60]. Anderson and Soden [60] pointed out that peer interaction is important for developing critical thinking skills.

In line with previous research [61], we identified the need for infrastructure, such as internet or Wi-Fi access, for technological tools to work properly [48]. Previous research has shown that simply setting up a technological tool, such as an e-learning environment, is not sufficient to support the development of critical thinking; resources, careful planning, and implementation are also required [59]. Our results indicate that technological tools were set up by educational institutions [48-51] and supervised mostly by lecturers [48,49,51] and that students were provided with regular feedback [48-51].

Our findings also show that technological tools are to varying degrees customized for the context in which they are used [48-51]. Students have called for bespoke technological tools [17]; however, as O’Connor and Andrews [17,61] found, most technological tools, such as mobile apps used in nursing education, are generic and not customized for the specific needs of nursing students. This may be particularly relevant if the aim of technological tools is to stimulate the development of critical thinking. However, if customized technological tools are used, they must be developed collaboratively with nursing students to ensure that their functionality addresses the specific context of nursing education and clinical practice [17].

We observed that technology-supported guidance models can cause stress that can influence the development of critical thinking [48,50]. O’Connor and Andrews [61] pointed out that technological tools have many advantages, but some students may experience them negatively, causing stress. Upadhyaya [62] described the stress caused by technological tools as *technostress*, which in the worst case may negatively influence students’ academic productivity.

Regarding the primary outcome, we found various usages of the term *critical thinking* and its synonyms, confirming earlier research. As Mundy and Denham [63] and Andreou et al [64] pointed out, there is no consistent agreement among educators or students in the understanding of critical thinking and the approaches that may support its development, which may pose a challenge in implementing technology-supported guidance models.

### Strengths and Limitations

This mixed method systematic review has several strengths. We conducted a comprehensive literature search and pairs of independent researchers appraised the quality of the resulting articles. We then conducted thematic synthesis with textual coding, with all authors coding the same texts, and the intercoder reliability was calculated to ensure the integrity of the results. Other strengths of this review include the inclusion of newer articles and the use of innovative contemporary technological tools in all included articles.

The primary outcome was critical thinking among nursing students in clinical practice. In line with our inclusion criteria and the definition of critical thinking according to Facione [4], we found that critical thinking was described directly by the concept of critical thinking, by the use of other synonymous concepts, or by a broader description.

All the included studies used some kind of intervention or technique to stimulate the development of critical thinking, although the aims of these interventions or techniques related to the development of critical thinking may be implied. Consequently, the included studies met the inclusion or exclusion criteria for this mixed methods review.

We followed the principles of thematic synthesis outlined by Thomas and Harden [43]. However, the researchers [43] did not use a traditional codebook in their approach to thematic synthesis; instead, they used a diagram of relationships among the descriptive themes, which was created by the EPPI Reviewer software [65]. As this study did not use EPPI Reviewer, we created a traditional codebook with code definitions to facilitate the coding process.

Thomas and Harden [43] did not outline a process for calculating intercoder reliability, but the authors of this study built a thematic synthesis on thematic analysis. Braun and Clarke [66] note that calculating intercoder reliability is an important step to ensure the quality and integrity of the coding process.

The initial result of the intercoder reliability calculation among all the authors indicated only fair agreement; however, that calculation clarified the need for further analysis, abstraction, and reduction of codes and themes, leading to a more robust coding process and result. The final intercoder reliability of analytical themes between the first and last authors indicates substantial agreement.

The main limitation of this systematic review is the quality of the included articles, all of which lacked detailed descriptions of technology-supported guidance models and research methods. However, we chose to include all studies in the synthesis, regardless of their methodological quality. One could argue that this decision weakened the final review, but our rationale was that the included studies uncovered a potential research gap, pointing to areas that merit exploration in future research, with a focus on methodological rigor. We were also able to identify valuable experiences with technology-supported guidance models that could both inspire and strengthen future research and the design of guidance models.

We also used a search strategy that identified several studies in Chinese and Korean that were potentially relevant to the aim of the review, but which, because of the language barrier, had to be excluded. Thus, we may have missed eligible studies that were unavailable in English.

Regarding other limitations, we tried on the basis of earlier research to identify all possible synonyms of the term *critical thinking*, which were used in the literature search and screening process. However, because of the multidimensional nature of critical thinking, we may have missed some terms that authors used to describe critical thinking, which may have influenced the search strategy and, consequently, the identification of potential articles.

### Conclusions

In nursing education, a few technology-supported guidance models that vary in setup and organization are used to stimulate the development of critical thinking in the context of clinical practice. The main characteristic of these models is the combination of instructional strategies with the active use of technological tools during guidance. The type of technological tools and how they are used in these guidance models vary across models. Thus, when using technology-supported guidance models, one should consider the underlying technological, instructional, and learning strategies incorporated in such models, as well as their suitability for intended use.

The main facilitators of these technology-supported guidance models are lecturers and nurse preceptors, who play an important role in nursing students’ guidance and support the development of critical thinking.

Technological tools themselves can be the primary facilitators of these guidance models, enabling the development of critical thinking. However, technology-supported guidance models can also cause stress, which may negatively affect the development of critical thinking skills among nursing students.

We do not have sufficient data nor it is the aim of this mixed method review to conclude with which technology-supported guidance model is superior or inferior in relation to supporting the development of critical thinking.

The findings of this mixed methods systematic review are relevant to the future development of technology-supported guidance models that support the development of critical thinking among nursing students in clinical practice. However, because of the quality of the included studies, we recommend that our results be used only as inspiration for further research or in designing new technology-supported guidance models.

## Appendix

Multimedia Appendix 1 PRISMA (Preferred Reporting Items for Systematic Reviews and Meta-Analyses) 2020 checklist.

Multimedia Appendix 2 Deviations from study protocol.

Multimedia Appendix 3 Example of the MEDLINE search strategy applied to all databases in the search strategy.

Multimedia Appendix 4 Study characteristics and methodological quality of the included studies.

Multimedia Appendix 5 Joanna Briggs Institute checklist for qualitative research—article 1.

Multimedia Appendix 6 Mixed Methods Appraisal Tool—article 2.

Multimedia Appendix 7 Joanna Briggs Institute checklist for qualitative research—article 3.

Multimedia Appendix 8 Joanna Briggs Institute checklist for quasi-experimental studies—article 4.

## Abbreviations

JBI: Joanna Briggs Institute
PRISMA: Preferred Reporting Items for Systematic Reviews and Meta-Analyses
