# Supplementary material for: Technology-Supported Guidance Models Stimulating the Development of Critical Thinking in Clinical Practice: Mixed Methods Systematic Review
Source: JMIR Nurs. 2022 Jun 7;5(1):e37380. doi: 10.2196/37380 (PMC9214617; doi:10.2196/37380)
Supplement: Multimedia Appendix 2 [file nursing_v5i1e37380_app2.pdf]

Multimedia Appendix 2. Deviations from the study protocol

Planned approach described in the protocol	Type, description and justification of the deviation from protocol
We planned to use EndNote (Clarivate) to manage records.	Type of deviation: clarification/change<br>We used Paperpile (Paperpile, LLC) instead of Endnote (Clarivate) because of its versatility in the storage and management of records.
We planned to use NVivo (QRS International) to store and synthesise data.	Type of deviation: clarification/change<br>We used MAXQDA (VERBI GmbH) instead of NVivo (ORS International) because of its availability and usability.
We planned to use thematic synthesis.	Type of deviation: addition<br>In addition to thematic synthesis, all the authors co-coded the text segments to enable calculating the inter-coder reliability. This was done to strengthen our results.
We planned to extract population, phenomenon of interest, type of study, methods, context, time period, percentage or average (in descriptive studies) and significant and nonsignificant results (in analytical studies). Furthermore, in qualitative studies, we planned to extract themes and subthemes with, for example, supporting quotations from the participants.	Type of deviation: addition<br>The data extraction also yielded the studies' country of origin and date of publication. We decided to include them to establish a better understanding of the background data in the articles and to strengthen our analysis.
We did not plan to calculate inter-coder reliability.	Type of deviation: addition<br>We included the calculation of inter-coder reliability to strengthen the coding process and the overall synthesis.

(Table adapted from Kinnear)
