# Supplementary material for: Technology-Supported Guidance Models Stimulating the Development of Critical Thinking in Clinical Practice: Mixed Methods Systematic Review
Source: JMIR Nurs. 2022 Jun 7;5(1):e37380. doi: 10.2196/37380 (PMC9214617; doi:10.2196/37380)
Supplement: Multimedia Appendix 3 [file nursing_v5i1e37380_app3.pdf]

Multimedia Appendix 3. Example of the MEDLINE search strategy applied to all databases in the search strategy

Database(s): Ovid MEDLINE(R) and Epub Ahead of Print, In-Process & Other Non-Indexed Citations and Daily 1946 to October 12, 2020

Search Strategy:

#	Searches	Results
1	((nurse or nursing or nurses) adj5 (student* or education or undergrad* or under-grad* or bachelor* or baccalaur* or preregistration or preregistration)).tw,kf.	54371
2	education, nursing/	32842
3	education, nursing, baccalaureate/	18254
4	Students, Nursing/	24711
5	Nursing Education Research/	8902
6	1 or 2 or 3 or 4 or 5	89716
7	(student* adj3 (practic* or practis* or placement* supervis* or clerkship* or apprenticeship*)).tw,kf.	8614
8	(clinic* adj3 (practic* or practis* or placement* or supervis* clerkship* or apprenticeship* or setting* or pedagog* or teach* or learn* or educat* or training*)).tw,kf.	357247
9	coach*.tw,kf.	16501
10	mentor*.tw,kf.	17740
11	preceptor*.tw,kf.	4387
12	clinical clerkship/	5142
13	mentoring/	1869
14	Mentors/	10901
15	Preceptorship/	5140
16	7 or 8 or 9 or 10 or 11 or 12 or 13 or 14	404474
17	Telecommunications/	4837
18	Telephone/	11885
19	Cell phone/	8637
20	Videoconferencing/	1626
21	Webcasts as topic/	348
22	Wireless technology/	3627
23	Text messaging/	3029
24	Technology/	9672
25	Educational technology/	1529
26	Mobile Applications/	6371
27	Computers, handheld/	3645
28	Smartphone/	4750
29	Internet/	73607
30	(mobile* or phone* or phoning or telephon* or cellphon* or smartphon* or iphon* or android*).tw,kf.	209604
31	electronic*.tw,kf.	298097
32	digital*.tw,kf.	158169
33	video*.tw,kf.	138815
34	web*.tw,kf.	176909
35	internet.tw,kf.	59156
36	online.tw,kf.	133179

37	app*1.tw,kf.	39987
38	app-base*.tw,kf.	717
39	telecommunicat*.tw,kf.	4739
40	tele-communicat*.tw,kf.	14
41	tablet*.tw,kf.	57355
42	((portable or handheld or hand-held) adj1 computer*).tw,kf.	1008
43	etutor*.tw,kf.	2
44	e-tutor*.tw,kf.	10
45	((short or text or instant) adj1 messag*).tw,kf.	6067
46	texting.tw,kf.	1038
47	sms.tw,kf.	6690
48	eportfolio*.tw,kf.	61
49	e-portfolio*.tw,kf.	141
50	(wireless adj2 communicat*).tw,kf.	2003
51	pda.tw,kf.	13037
52	personal digital assistant*.tw,kf.	1041
53	"virtual session*".tw,kf.	17
54	17 or 18 or 19 or 20 or 21 or 22 or 23 or 24 or 25 or 26 or 27 or 28 or 29 or 30 or 31 or 32 or 33 or 34 or 35 or 36 or 37 or 38 or 39 or 40 or 41 or 42 or 43 or 44 or 45 or 46 or 47 or 48 or 49 or 50 or 51 or 52 or 53	1193131
55	6 and 16 and 54	1763
56	limit 55 to yr="2010 -Current"	1368
57	limit 56 to (comment or editorial or letter)	6
58	56 not 57	1362
