# Supplementary material for: Technology-Supported Guidance Models Stimulating the Development of Critical Thinking in Clinical Practice: Mixed Methods Systematic Review
Source: JMIR Nurs. 2022 Jun 7;5(1):e37380. doi: 10.2196/37380 (PMC9214617; doi:10.2196/37380)
Supplement: Multimedia Appendix 4 [file nursing_v5i1e37380_app4.pdf]

Study characteristics and methodological quality of included studies

Authors, (year and country)	Aim	Sample	Design	Data collection methods	Description of methodological quality	Results and conclusion
Mettiäinen, Sari; Vähämaa, Kristiina, (2013, Finland)	To study the use of a web-based discussion board (WBDB) as a supervision tool during nursing students' clinical practice.	25 3rd year nursing students, during 8 weeks of clinical practice.<br><br>Sex and age were not reported	Qualitative study.	Qualitative data collected from discussion forum.	The investigators did not describe their own role (e.g. teachers). Unclear if collected data were anonymized. Lack of design description, poorly described methods.	WBDB with lecture supervision and peer support stimulated nursing students' reflection, learning process and clinical skills. The authors conclude with that the WBDB could contribute to development of critical thinking.
Lai, Chin-Yuan; Wu, Cheng-Chih, (2016, Taiwan)	To test the effect of e-portfolio system (EPF) in nursing students' clinical practice. The authors measured critical thinking on a competence scale.	10 2nd year female nursing students, 21.3 years old in average during 3 weeks of clinical practice.	A mixed-methods study.	Quantitative data from self-rated and instructor rated scale questionnaires. Qualitative data from questionnaires with open ended questions.	No rationale presented for choice of design. No adequate interpretation of qualitative and quantitative data. Lack of adherence to quality criteria of chosen methods.	The EPF promoted nursing students self-regulated learning with professional progress in both theory and practice (clinical competence). Nursing students' positive attitudes were connected to the use of the system, but nursing students experience also occasional stress and technical difficulties. Improvement of critical thinking skills, as a part of a competence was observed (2.7 score at week 1 to 4.3 at week 3 at (P< .001).
Lai, Chin-Yuan; Yen, Yung-Chin (2018, Taiwan)	To illustrate how a cognitive apprenticeship model (CAM) could be applied to enhance clinical placement experiences for nursing students through the use of mobile devices.	8 4th grade female nursing students, 19.2 years old in average (from a 5-year nursing programme) during 3 weeks of clinical practice.	Qualitative study.	Focus group interviews.	The authors refer to their findings as effect, but what they present are experiences from nursing students and instructors.	CAM delivered by mobile devices promoted processes of reflection coaching, scaffolding and articulation among nursing students, which the authors considers as a way of promoting critical thinking.
Wu, Ting-Ting; Huang, Yueh-Min; Su, Chen-Ying; Chang, Lei; Lu, Yi Chen, (2018, Taiwain)	To test an e-book system integrating project-based learning and authentic learning during a community health nursing practice.	64 nursing students during 3 weeks of clinical practice.<br><br>Sex, age and year of study were not reported	Quasi experimental study.	Quantitative data with an assessment scale of learning effectiveness.	The background characteristics of the students are lacking, and the statistical analysis is unclear.	The e-book system increased nursing students learning interest motivation and improved nursing students learning effectiveness. The authors measured critical thinking among participants, but did not provide any results specifically on critical thinking.
