# Supplementary material for: Technology-Supported Guidance Models Stimulating the Development of Critical Thinking in Clinical Practice: Mixed Methods Systematic Review
Source: JMIR Nurs. 2022 Jun 7;5(1):e37380. doi: 10.2196/37380 (PMC9214617; doi:10.2196/37380)
Supplement: Multimedia Appendix 8 [file nursing_v5i1e37380_app8.pdf]

THE JOANNA BRIGGS INSTITUTE
Better evidence. Better outcomes.

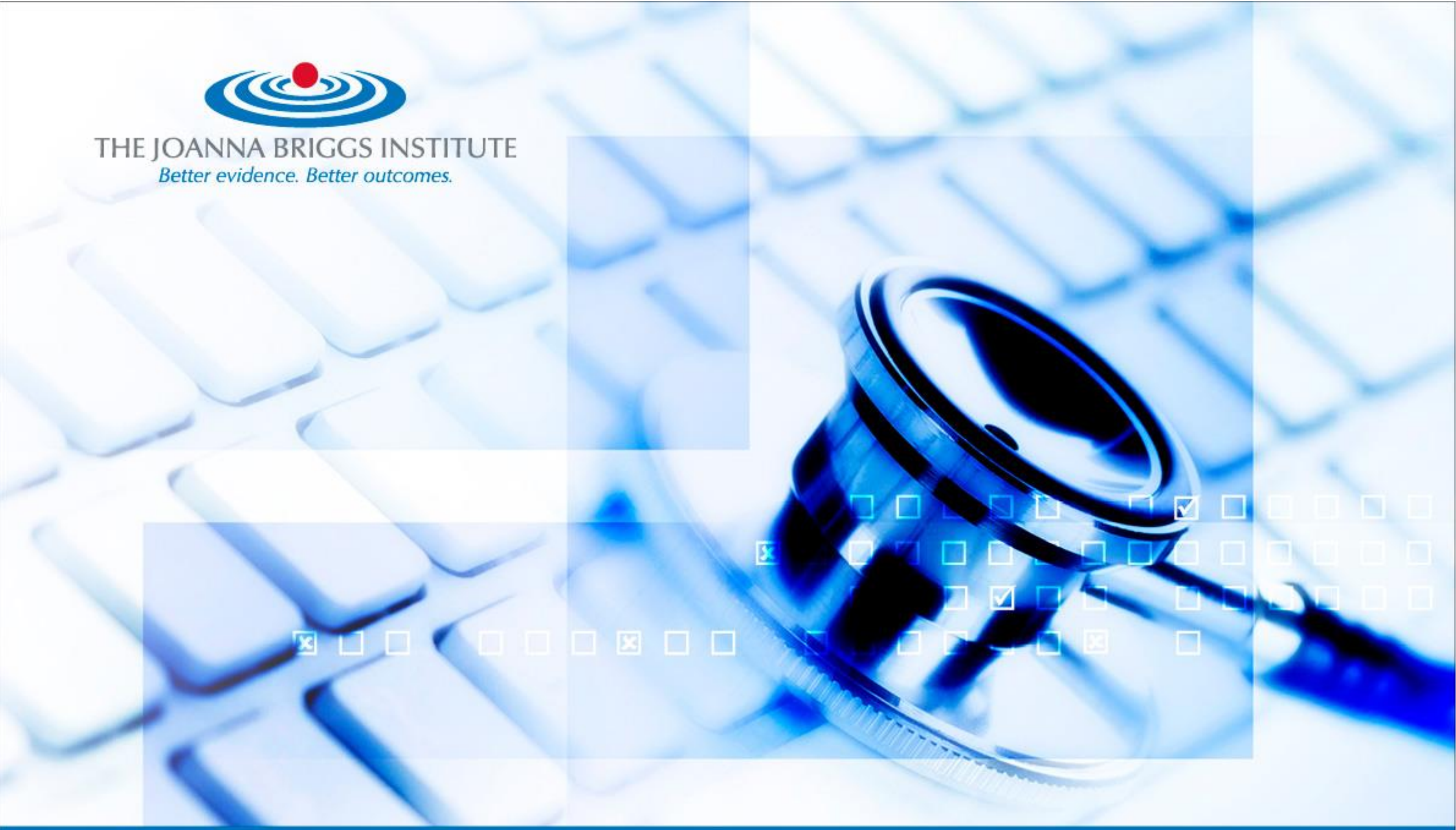

The Joanna Briggs Institute Critical Appraisal tools
for use in JBI Systematic Reviews

Checklist for Quasi-Experimental Studies

(non-randomized experimental studies)

http://joannabriggs.org/research/critical-appraisal-tools.html

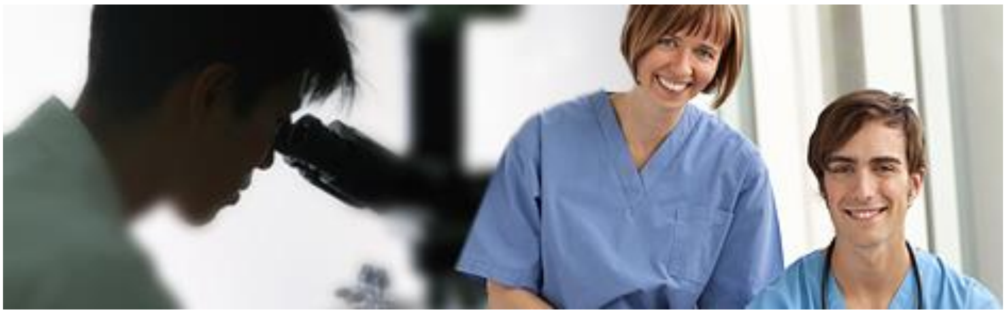

The Joanna Briggs Institute

Introduction

The Joanna Briggs Institute (JBI) is an international, membership based research and development organization within the Faculty of Health Sciences at the University of Adelaide. The Institute specializes in promoting and supporting evidence-based healthcare by providing access to resources for professionals in nursing, midwifery, medicine, and allied health. With over 80 collaborating centres and entities, servicing over 90 countries, the Institute is a recognized global leader in evidence-based healthcare.

JBI Systematic Reviews

The core of evidence synthesis is the systematic review of literature of a particular intervention, condition or issue. The systematic review is essentially an analysis of the available literature (that is, evidence) and a judgment of the effectiveness or otherwise of a practice, involving a series of complex steps. The JBI takes a particular view on what counts as evidence and the methods utilized to synthesize those different types of evidence. In line with this broader view of evidence, the Institute has developed theories, methodologies and rigorous processes for the critical appraisal and synthesis of these diverse forms of evidence in order to aid in clinical decision-making in health care. There now exists JBI guidance for conducting reviews of effectiveness research, qualitative research, prevalence/incidence, etiology/risk, economic evaluations, text/opinion, diagnostic test accuracy, mixed-methods, umbrella reviews and scoping reviews. Further information regarding JBI systematic reviews can be found in the JBI Reviewer's Manual on our website.

JBI Critical Appraisal Tools

All systematic reviews incorporate a process of critique or appraisal of the research evidence. The purpose of this appraisal is to assess the methodological quality of a study and to determine the extent to which a study has addressed the possibility of bias in its design, conduct and analysis. All papers selected for inclusion in the systematic review (that is – those that meet the inclusion criteria described in the protocol) need to be subjected to rigorous appraisal by two critical appraisers. The results of this appraisal can then be used to inform synthesis and interpretation of the results of the study. JBI Critical appraisal tools have been developed by the JBI and collaborators and approved by the JBI Scientific Committee following extensive peer review. Although designed for use in systematic reviews, JBI critical appraisal tools can also be used when creating Critically Appraised Topics (CAT), in journal clubs and as an educational tool.

© Joanna Briggs Institute 2017

THE JOANNA BRIGGS INSTITUTE

JBI Critical Appraisal Checklist for Quasi-Experimental Studies (non-randomized experimental studies)

Reviewer Mariann Fossum Date 19.01.2021

Author Wu, T.-T., Huang, Y.-M., Su, C.-Y., Chang, L., & Lu, Y. C. Year 2018 Record Number 3

		Yes	No	Unclear	Not applicable
1.	Is it clear in the study what is the 'cause' and what is the 'effect' (i.e. there is no confusion about which variable comes first)?	☐	☒	☐	☐
2.	Were the participants included in any comparisons similar?	☐	☐	☒	☐
3.	Were the participants included in any comparisons receiving similar treatment/care, other than the exposure or intervention of interest?	☐	☐	☒	☐
4.	Was there a control group?	☒	☐	☐	☐
5.	Were there multiple measurements of the outcome both pre and post the intervention/exposure?	☐	☒	☐	☐
6.	Was follow up complete and if not, were differences between groups in terms of their follow up adequately described and analyzed?	☐	☒	☐	☐
7.	Were the outcomes of participants included in any comparisons measured in the same way?	☒	☐	☐	☐
8.	Were outcomes measured in a reliable way?	☐	☐	☒	☐
9.	Was appropriate statistical analysis used?	☐	☒	☐	☐

Overall appraisal: Include ☐ Exclude ☒ Seek further info ☐

Comments (Including reason for exclusion)

The background charateristics of the students are lacking, and the statistical analysis unclear.

THE JOANNA BRIGGS INSTITUTE

Explanation for the critical appraisal tool for Quasi-Experimental Studies (experimental studies without random allocation)

How to cite: Tufanaru C, Munn Z, Aromataris E, Campbell J, Hopp L. Chapter 3: Systematic reviews of effectiveness. In: Aromataris E, Munn Z (Editors). *Joanna Briggs Institute Reviewer's Manual.* The Joanna Briggs Institute, 2017. Available from https://reviewersmanual.joannabriggs.org/

Critical Appraisal Tool for Quasi-Experimental Studies (experimental studies without random allocation)

Answers: Yes, No, Unclear or Not Applicable

1. **Is it clear in the study what is the 'cause' and what is the 'effect' (i.e. there is no confusion about which variable comes first)?**

Ambiguity with regards to the temporal relationship of variables constitutes a threat to the internal validity of a study exploring causal relationships. The 'cause' (the independent variable, that is, the treatment or intervention of interest) should occur in time before the explored 'effect' (the dependent variable, which is the effect or outcome of interest). Check if it is clear which variable is manipulated as a potential cause. Check if it is clear which variable is measured as the effect of the potential cause. Is it clear that the 'cause' was manipulated before the occurrence of the 'effect'?

2. Were the participants included in any comparisons similar?

The differences between participants included in compared groups constitute a threat to the internal validity of a study exploring causal relationships. If there are differences between participants included in compared groups there is a risk of selection bias. If there are differences between participants included in the compared groups maybe the 'effect' cannot be attributed to the potential 'cause', as maybe it is plausible that the 'effect' may be explained by the differences between participants, that is, by selection bias. Check the characteristics reported for participants. Are the participants from the compared groups similar with regards to the characteristics that may explain the effect even in the absence of the 'cause', for example, age, severity of the disease, stage of the disease, co-existing conditions and so on? *[NOTE: In one single group pre-test/post-test studies where the patients are the same (the same one group) in any pre-post comparisons, the answer to this question should be 'yes.']*

3. Were the participants included in any comparisons receiving similar treatment/care, other than the exposure or intervention of interest?

In order to attribute the 'effect' to the 'cause' (the exposure or intervention of interest), assuming that there is no selection bias, there should be no other difference between the groups in terms of treatments or care received, other than the manipulated 'cause' (the intervention of

interest). If there are other exposures or treatments occurring in the same time with the 'cause', other than the intervention of interest, then potentially the 'effect' cannot be attributed to the intervention of interest, as it is plausible that the 'effect' may be explained by other exposures or treatments, other than the intervention of interest, occurring in the same time with the intervention of interest. Check the reported exposures or interventions received by the compared groups. Are there other exposures or treatments occurring in the same time with the intervention of interest? Is it plausible that the 'effect' may be explained by other exposures or treatments occurring in the same time with the intervention of interest?

4. **Was there a control group?**

Control groups offer the conditions to explore what would have happened with groups exposed to other different treatments, other than to the potential 'cause' (the intervention of interest). The comparison of the treated group (the group exposed to the examined 'cause', that is, the group receiving the intervention of interest) with such other groups strengthens the examination of the causal plausibility. The validity of causal inferences is strengthened in studies with at least one independent control group compared to studies without an independent control group. Check if there are independent, separate groups, used as control groups in the study. *[Note: The control group should be an independent, separate control group, not the pre-test group in a single group pre-test post-test design.]*

5. Were there multiple measurements of the outcome both pre and post the intervention/exposure?

In order to show that there is a change in the outcome (the 'effect') as a result of the intervention/treatment (the 'cause') it is necessary to compare the results of measurement before and after the intervention/treatment. If there is no measurement before the treatment and only measurement after the treatment is available it is not known if there is a change after the treatment compared to before the treatment. If multiple measurements are collected before the intervention/treatment is implemented then it is possible to explore the plausibility of alternative explanations other than the proposed 'cause' (the intervention of interest) for the observed 'effect', such as the naturally occurring changes in the absence of the 'cause', and changes of high (or low) scores towards less extreme values even in the absence of the 'cause' (sometimes called regression to the mean). If multiple measurements are collected after the intervention/treatment is implemented it is possible to explore the changes of the 'effect' in time in each group and to compare these changes across the groups. Check if measurements were collected before the intervention of interest was implemented. Were there multiple pre-test measurements? Check if measurements were collected after the intervention of interest was implemented. Were there multiple post-test measurements?

6. Was follow up complete and if not, were differences between groups in terms of their follow up adequately described and analyzed?

If there are differences with regards to the loss to follow up between the compared groups these differences represent a threat to the internal validity of a study exploring causal effects as these differences may provide a plausible alternative explanation for the observed ‘effect’ even in the absence of the ‘cause’ (the treatment or exposure of interest). Check if there were differences with regards to the loss to follow up between the compared groups. If follow up was incomplete (that is, there is incomplete information on all participants), examine the reported details about the strategies used in order to address incomplete follow up, such as descriptions of loss to follow up (absolute numbers; proportions; reasons for loss to follow up; patterns of loss to follow up) and impact analyses (the analyses of the impact of loss to follow up on results). Was there a description of the incomplete follow up (number of participants and the specific reasons for loss to follow up)? If there are differences between groups with regards to the loss to follow up, was there an analysis of patterns of loss to follow up? If there are differences between the groups with regards to the loss to follow up, was there an analysis of the impact of the loss to follow up on the results?

7. Were the outcomes of participants included in any comparisons measured in the same way?
If the outcome (the ‘effect’) is not measured in the same way in the compared groups there is a threat to the internal validity of a study exploring a causal relationship as the differences in outcome measurements may be confused with an effect of the treatment or intervention of interest (the ‘cause’). Check if the outcomes were measured in the same way. Same instrument or scale used? Same measurement timing? Same measurement procedures and instructions?

8. Were outcomes measured in a reliable way?

Unreliability of outcome measurements is one threat that weakens the validity of inferences about the statistical relationship between the ‘cause’ and the ‘effect’ estimated in a study exploring causal effects. Unreliability of outcome measurements is one of different plausible explanations for errors of statistical inference with regards to the existence and the magnitude of the effect determined by the treatment (‘cause’). Check the details about the reliability of measurement such as the number of raters, training of raters, the intra-rater reliability, and the inter-raters reliability within the study (not to external sources). This question is about the reliability of the measurement performed in the study, it is not about the validity of the measurement instruments/scales used in the study. *[Note: Two other important threats that weaken the validity of inferences about the statistical relationship between the ‘cause’ and the ‘effect’ are low statistical power and the violation of the assumptions of statistical tests. These other threats are not explored within Question 8, these are explored within Question 9.]*

9. Was appropriate statistical analysis used?

Inappropriate statistical analysis may cause errors of statistical inference with regards to the existence and the magnitude of the effect determined by the treatment (‘cause’). Low statistical power and the violation of the assumptions of statistical tests are two important threats that weakens the validity of inferences about the statistical relationship between the ‘cause’ and the ‘effect’. Check the following aspects: if the assumptions of statistical tests were respected; if appropriate statistical power analysis was performed; if appropriate effect sizes were used; if appropriate statistical procedures or methods were used given the number and type of dependent and independent variables, the number of study groups, the nature of the relationship between the groups (independent or dependent groups), and the objectives of statistical analysis (association between variables; prediction; survival analysis etc.).

© Joanna Briggs Institute 2017
